# Fertility preservation service for children and young adults at high risk of infertility; the hub and spoke model

**DOI:** 10.1007/s00383-024-05770-5

**Published:** 2024-07-19

**Authors:** Harmit Ghattaura, Jill Davies, Christian M. Becker, Sheila Lane, Kokila Lakhoo

**Affiliations:** 1 Department of Paediatric Surgery, The Children’s Hospital, Oxford University Hospitals NHS Trust, Headley Way, Headington, Oxford OX3 9DU UK; 2 Oxford Cell & Tissue Biobank, The John Radcliffe Hospital, Oxford University Hospitals NHS Trust, Headley Way, Headington, Oxford OX3 9DU UK; 3 Nuffield Department of Women’s and Reproductive Health, Medical Sciences Division, University of Oxford, Oxford, OX3 9DU UK; 4 Department of Paediatric Oncology, The Children’s Hospital, Oxford University Hospitals NHS Trust, Headley Way, Headington, Oxford OX3 9DU UK; 5 Nuffield Department of Surgical Sciences, University of Oxford, Level 6, John Radcliffe Hospital, Headington, Oxford OX3 9DU UK

**Keywords:** Fertility Preservation, Paediatric, Oncology, Hub and Spoke

## Abstract

**Purpose:**

To evaluate the evolution of fertility preservation surgery in children and young adults at high risk of infertility from a single centre to a networked ‘Hub and Spoke’ service.

**Methods:**

A case note review of patients referred for ovarian or testicular cryopreservation between Jan 2013 and Dec 2023. Demographic data, procurement numbers, and site of procurement were collected. Specialist feedback was obtained to identify the challenges faced.

**Results:**

Over time, the number of referrals increased from 4 to 349 patients per year with the number of Spoke centres rising to 36 ovarian and 16 testicular. In 2013–2014; 100% of procurement was ovarian as compared to 2023; 51% ovarian, 49% testicular. Of the 395 referrals in 2021, 81% (n = 319) went on to have procurement and storage of tissue. Between 2013 and 2016, 96% of cases were performed at the Hub. In 2023, 53/349 (15%) cases were performed at the Hub with the remaining 296 (85%) procured at Spoke sites. Surgical issues such as access to theatre, variation and availability of surgical equipment, thermal injury to ovarian tissue and variation in the size of the testicular specimen were identified.

**Conclusion:**

The Hub and Spoke model successfully delivers treatment to patients close to home as safely possible within their local treatment centre.

## Background

Cryopreservation is the use of very low temperatures to preserve structurally intact living cells and tissues. Children and young adults who are undergoing gonadotoxic treatments which include chemotherapy, pelvic, or total body irradiation maybe offered cryopreservation of their gonadal tissue. There is now considerable emerging evidence as to the proven feasibility of the cryopreservation of ovarian tissue resulting in successful live births after re-implantation. A documented 130 live births have been reported with evidence that there is no increased morbidity compared to the general population using this technique [1, 2]. Alongside the uses discussed within this article, human ovarian tissue cryopreservation may also theoretically be applied as an alternative to postpone menopause in healthy women [3].

A publication in 2019 described 3 fertility preservation networks and discussed these in depth. These 3 are grouped into ‘centralised’, ‘centralised as well as decentralised’, and ‘completely decentralised’. The article provides an insight into the variability of the organisation of cryopreservation services. The publication also emphasises additional aspects including the need for educational, political, and financial support required to maintain a well-organised network. Furthermore, the Oncofertility Consortium described additional stakeholders of lawyers, insurance experts, religious leaders, and bioethicists. Data collection through prospective registries is also discussed and of paramount importance. Our article therefore provides further insight into the wider world of fertility preservation across all age groups [4].

The United Kingdom Children’s Cancer and Leukaemia Group (UK CCLG) oncofertility consensus document outlines the assessment of fertility and fertility preservation options offered to children within the UK who undergo gonadotoxic treatments [5]. All children deemed as high risk who wish to have gonadal tissue harvested will undergo an individual assessment and decide whether to proceed.

Over the past decade, as awareness of the availability of fertility treatment for children and young adults has increased, there has been an exponential increase in demand for our services. The Hub and Spoke model has been used in a number of settings to arrange the delivery of services across a wide area. Traditionally, the Hub is an establishment that offers a full array of services which is complemented by secondary establishments that also offer part of the service. Although this has its origins in business, several articles exist highlighting its use in the healthcare setting [6–9].

We present the model through which we have been able to offer all children across England, Wales and Northern Ireland a fertility cryopreservation service. We work collaboratively with colleagues in Edinburgh to ensure treatment across the whole of the UK. As tissue is collected in a number of different centres, we aim to outline the way we hope to standardise and optimise techniques to improve the quality of the specimen and provide our patients with the best chances of successful fertility preservation.

## Method

We conducted a review of cases to describe the evolution and development of the Hub and Spoke model across the time period Jan 2013–Dec 2023. At our institution, we offer a service for both ovarian and testicular cryopreservation. We collected data about the patient demographics alongside their diagnosis at referral. We also collected data for those who went onto procurement including whether this was performed at a Hub or a Spoke site. Furthermore, after discussion with members of the Hub cryopreservation team and some of the spoke consultants we identified some of the current challenges faced and formulated ways that these could be tackled.

## Surgical technique

Ovarian harvesting is performed under general anaesthetic and in most cases, a single entire ovary is removed using haemostatic dissection. The tissue is then transported to the Oxford University Hospitals (OUH) Human tissue Authority (HTA) licenced Oxford Cell and Tissue Biobank (OCTB) where it is processed and cryopreserved.

For the harvesting of gonadal tissue in children at the Hub, a single incision laparoscopy technique (SILS) is used for the collection of ovarian tissue. This technique begins with a vertical trans-umbilical incision and insertion of the GelPoint® port. Laparoscopy is then performed and clinical photographs of both the ovaries are taken for the patient record. Often, unless other factors dictate a different approach, the right ovary is removed as it is easier to mobilise and is located away from the sigmoid colon. Factors affecting selection may include the size of the ovary, any evidence of disease and also proximity to the underlying pathology and delivery of gonadotoxic treatment such as pelvic radiotherapy. We prefer to harvest ovaries that are disease free and larger. An atraumatic grasper is used to provide countertraction whilst the Ligasure is used to perform dissection of the mesovarium between the ovary and the fallopian tube until the utero-ovarian ligament. The specimen is then delivered via the umbilical incision through the GelPoint® port. There is some evidence, within adult literature, that single port laparoscopy results in minimal trauma at delivery of the ovary and subsequent better quality of tissue for ovarian cryopreservation. The development of our surgical technique for ovarian harvesting has been described in depth in another article [10].

For testicular cryopreservation, we measure the testicles using an orchidometer pre-operatively. If applicable, the side of the larger testis is chosen and a transverse hemi-scrotal incision is made. The testis is delivered and the surgeon uses a knife to incise the tunica albuginea and harvest an ‘inverted wedge’ of tissue which is at least one-third of the volume of the testis. During this, the testis can be held between the thumb and the index finger with the cord compressed between the middle finger and palm of the hand. Once removed, the specimen is handed to the tissue bank technician who will confirm adequacy. The tunica albuginea is closed using a continuous interlocking haemostatic suture.

Our centre has developed the abovementioned techniques over years of gonadal harvesting with the aim of optimising the quality of the specimen. At Spoke centres, there is variation in practice and surgical technique and none use single incision laparoscopy for ovarian harvesting.

## Cryopreservation technique

Our Hub centre tissue bank has a standard protocol for the processing of ovarian and testicular tissue for cryopreservation. Harvesting is performed by selected surgeons during a dedicated cryopreservation list. Tissue is transferred to the Tissue Bank technician who must be present in the theatre.

During harvest at a spoke centre, the tissue bank technicians organise for validated temperature controlled and monitored boxes (DSX Diagnosach, Barcelona, Spain) to take all reagents and equipment to the procurement centres, using a contracted ambulance company. This box is handed over by the ambulance company to a named procurement centre co-ordinator, who has been trained by OCTB. They will then take it to theatre and collect the harvested tissue alongside blood samples (required for blood-borne virus screening) before handing this to the ambulance courier who remains waiting at the Spoke centre. This box is then transported directly to the OCTB by contracted ambulance couriers. Transit time for specimens is mapped and the ambulance couriers are equipped with blue lights to ensure specimens are always delivered in a timely manner. A member of the OCTB is always available, even out of hours, to collect the box and ensure there is no damage.

Tissue is then processed in the OCTB pharmaceutical manufacturing-compliant cleanroom. The quality is assessed by trained technical staff before being dissected into 1 mm thick strips (ovarian) or squares (testicular). Tissue is then immersed into cryovials containing 1 ml of cryomedia. Ovarian cryoprotectant (CPA) used is ethylene glycol (permeating) with sucrose (non-permeating) and testicular CPA used is dimethyl sulphoxide (DMSO) (permeating) with sucrose. The cryovials undergo tissue-specific cooling programmes and are stored in a vapour phase liquid nitrogen freezer at < –170’C until such a time that the patient wishes to use their tissue or until they no longer need it (e.g., after death).

## Results

### Model development

Our OCTB is a small tissue bank operating within Oxford University Hospital (OUH). We gained a license for ovarian tissue cryopreservation in 2013 and a license for cryopreservation of testicular tissue in 2015. During Phase 1, referrals for local patients were forwarded to the OUH clinical team for eligibility review. Procurement was undertaken by Hub paediatric and adult surgical teams and all tissue then cryopreserved and stored by OCTB under the OUH HTA Human Application license.

In the following years, referrals to the Hub for ovarian tissue cryopreservation and testicular tissue cryopreservation increased exponentially. If patients were travelling from a considerable distance, then they would travel to Oxford on the day before the operation and stay in local accommodation overnight. The harvesting of gonadal tissue for cryopreservation would often be performed with concomitant time-critical procedures including tunnelled central line insertion. Phase 2 was initiated in response to the increasing demands on the service and an HTA license extension was then gained by OCTB to set up control measures and attend procurement of tissue at a surgical centre near the patient (known as ‘Spoke’ centres).

During Phase 3 and in the absence of national funding for the service, the small team of OCTB technicians was unable to meet demands of the Spoke centres. Further approval was gained by OCTB to train local staff (co-ordinators) at procurement centres so they could attend procurement to perform OCTB-specified protocols for witnessing, packaging, and labelling procedures in accordance with HTA requirements. The OCTB also gained authorisation to provide and record training of key consultants for face-to-face consenting of patients at their local centre.

**Fig. 1:**
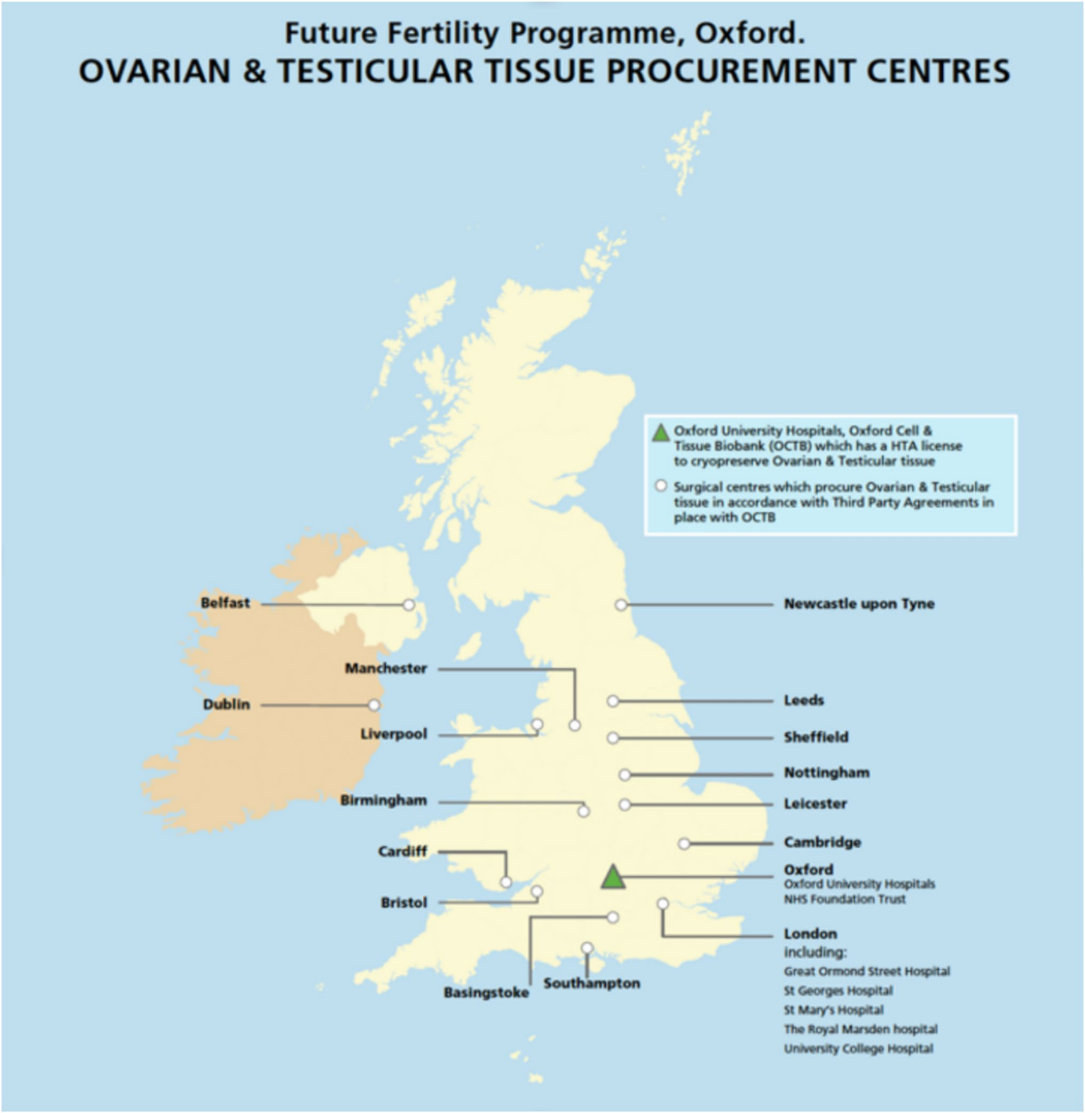
An illustration of the current Hub and Spoke Network

This allowed the patient to stay local but the gonadal tissue to travel. Once a patient within a Spoke centre was deemed eligible to undergo cryopreservation, they were referred to the Hub for a telemedical consultation. If the family/patient wished to proceed with reproductive tissue collection, a time and date were agreed for surgery and the OCTB-trained co-ordinator informed. If the co-ordinator could not attend due to sickness or absence, an OCTB technician would travel with the box to the Spoke to ensure the child received the treatment. The current network is shown in Fig. 1. Within Scotland, Edinburgh acts as the Hub for its own fertility preservation service.

### The Hub and Spoke model

**Fig. 2:**
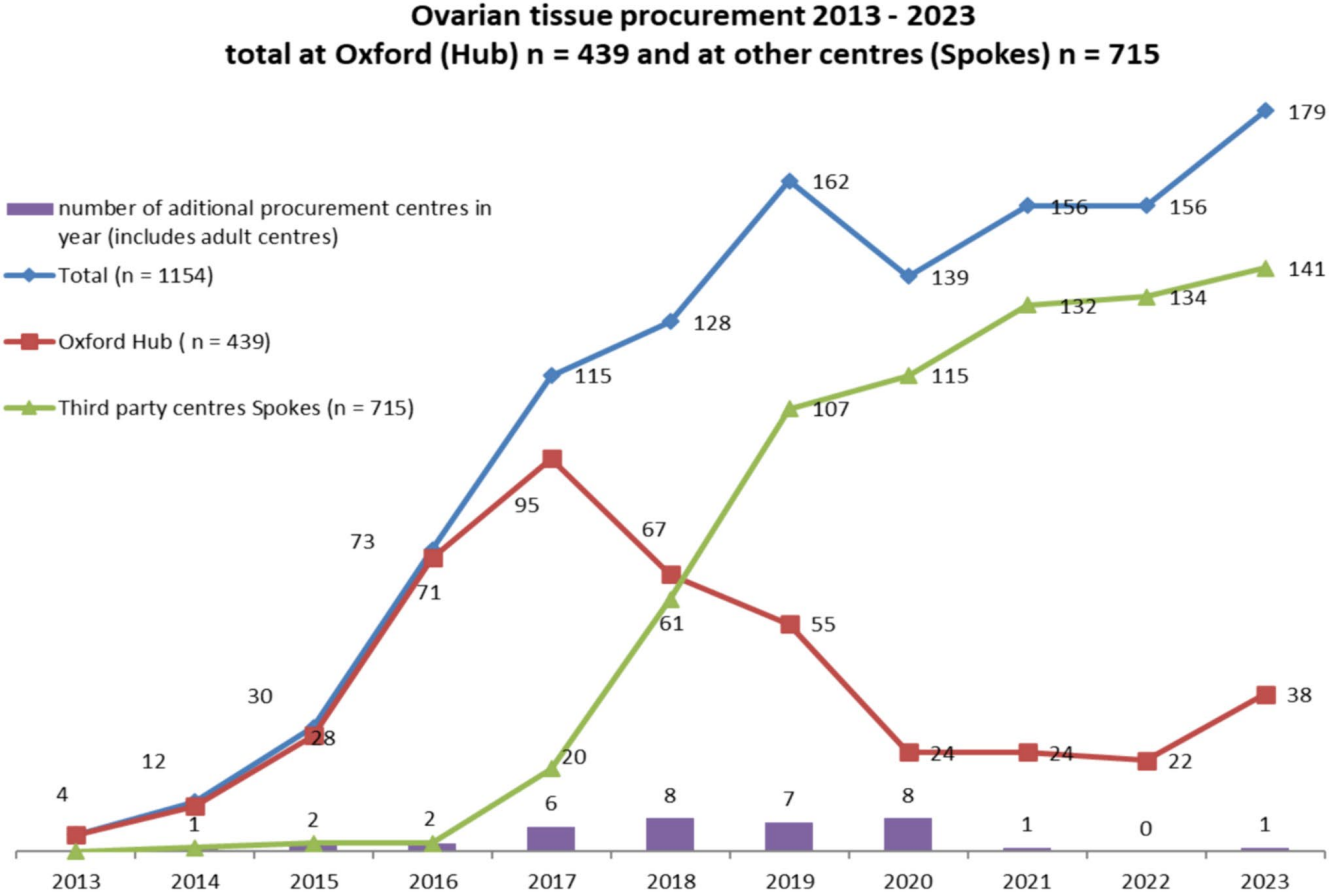
Trends in procurement numbers over time in Hub versus Spoke centre; Ovarian

**Fig. 3:**
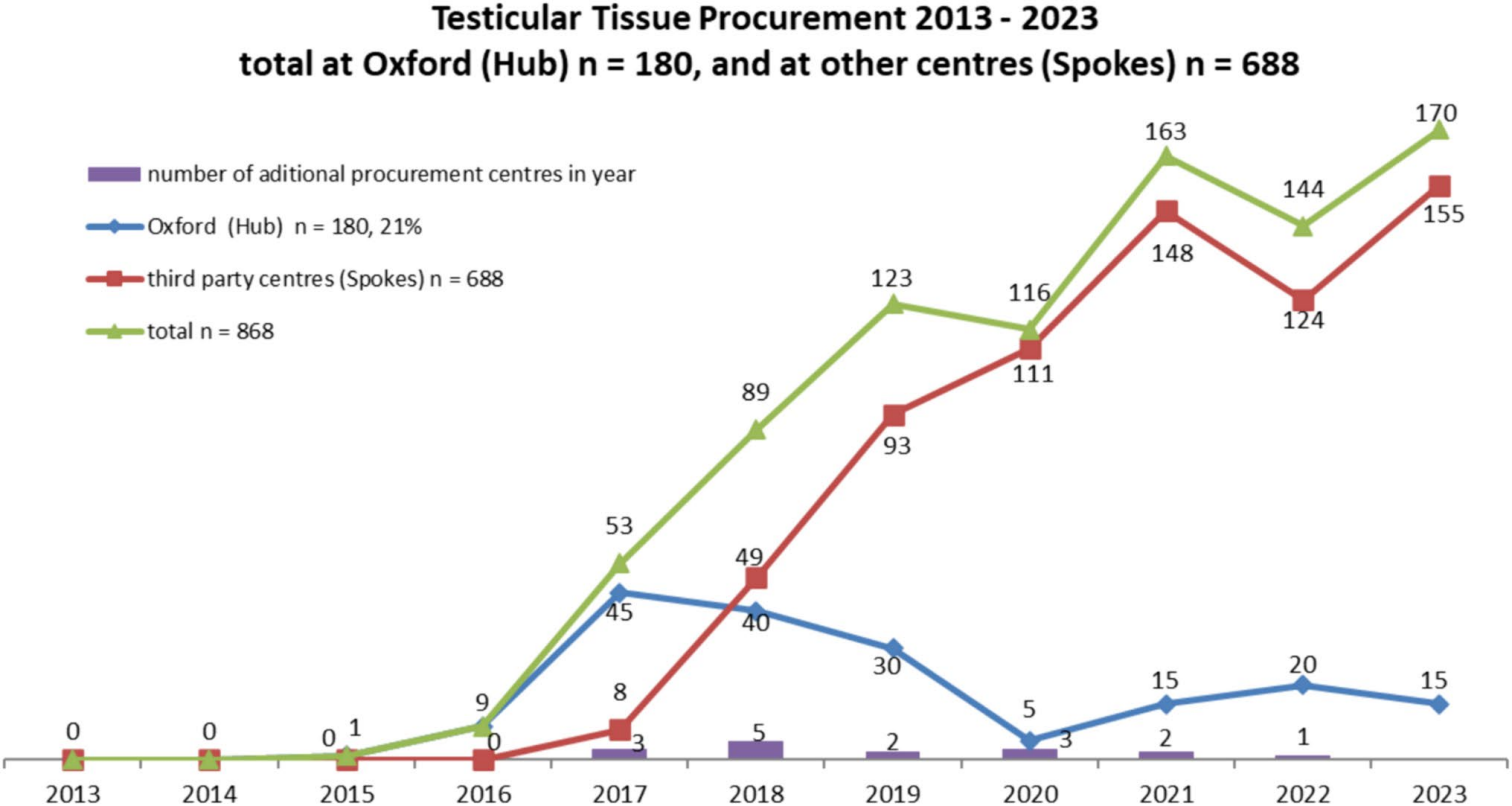
Trends in procurement numbers over time in Hub versus Spoke centre; Testicular

Over the study period, the number of referrals for cryopreservation increased from 4 to 349 patients per year. The first testicular cryopreservation was performed in 2015; which was immediately after the HTA awarded our testicular cryopreservation license. Over the study period, mean age of ovarian harvest was 11.87 years (5 weeks—39 years 9.5 months). The mean age at procurement of testicular tissue was 7.15 years (7 weeks—25 years 5 months). In 2013–2014; 100% were ovarian as compared to 2023; 51% ovarian, 49% testicular. Between 2013 and 2016, 96% of cases were performed at the Hub. In 2023, 53/349 (15%) cases were performed at the Hub with the remaining 296 (85%) procured at Spoke sites. Figures 2, 3 demonstrates the evolution of the model over time for procurement of both ovarian and testicular tissue.

**Fig. 4:**
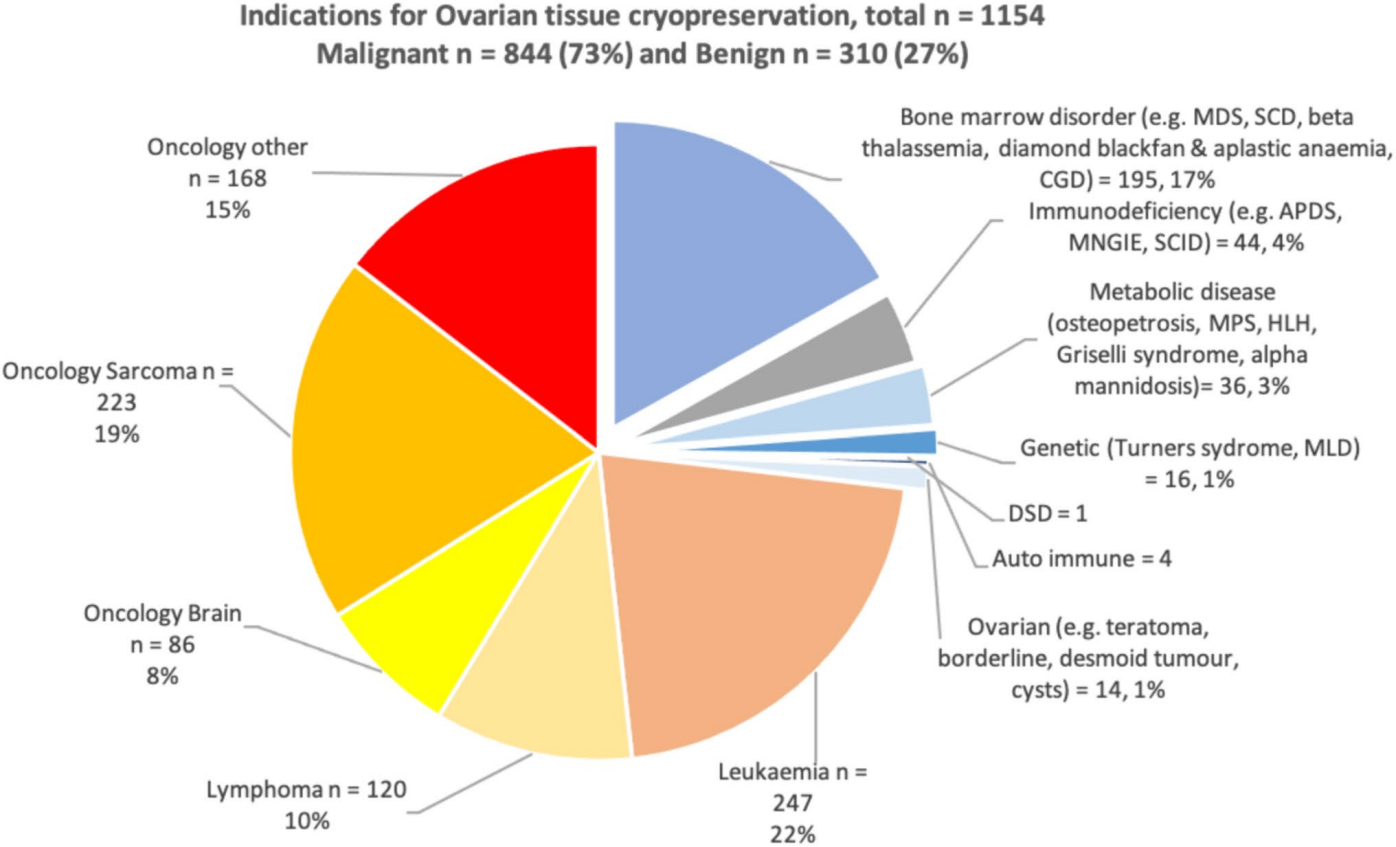
Indications for ovarian tissue cryopreservation by diagnosis over study period

**Fig. 5:**
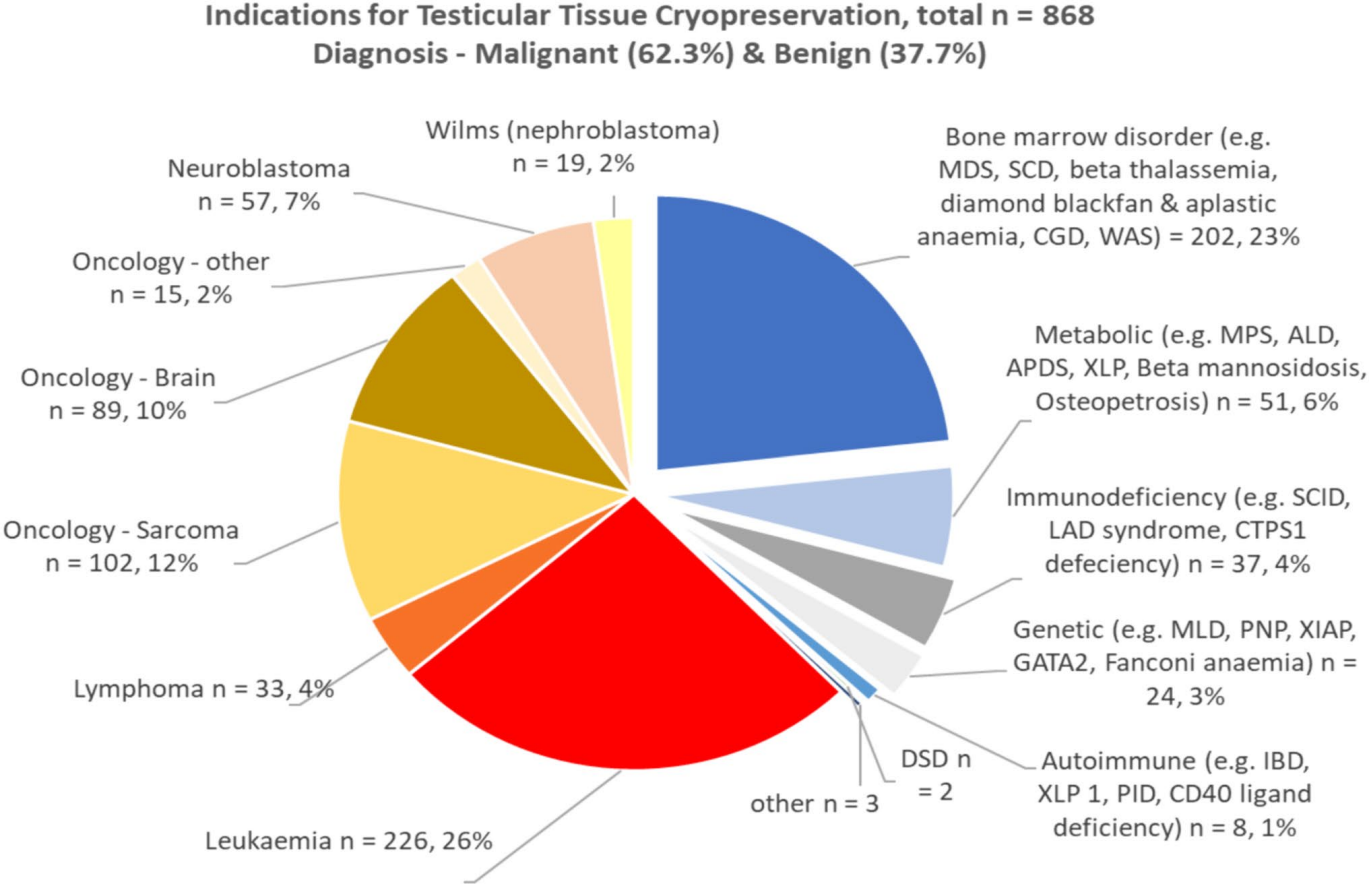
Indications for testicular tissue cryopreservation by diagnosis over study period

With respect to ovarian preservation, 27% had benign pathology whereas 73% had malignancy. With respect to testicular preservation, a considerably higher proportion of 38% had benign disease vs 62% who had malignant disease. As demonstrated in Figs. 4, 5, of the benign conditions, the most common indication for referral was bone marrow disorder including haemoglobinopathies. The top 3 malignant conditions resulting in referral were relapsed leukaemia, lymphoma, and sarcoma.

There were no problems associated with the transport of the specimens. The mean transit time measured from the time the box leaves the OCTB until the return was 6.5 h (range 2.5–16 h). All validated temperature-controlled boxes have been received intact with no concerns for damage. This technique has worked well since its inception and required no modifications.

Within our series, a total of 10 ovarian transplantations have been successfully performed with the aim of restoration of both fertility and hormonal function. The specimens were thawed by the tissue bank team who were present in theatre to prepare and hand over the specimen to be reimplanted by the gynaecological team. To date, testicular tissue has not been used to restore fertility although the final proof of concept has been published [11].

## Discussion

The Hub and Spoke model has proven to be extremely successful in allowing access to the cryopreservation service for children and young adults who are at high risk of infertility. The overriding argument for the utility of this model is that without it; it is highly likely that the Hub would not be able to cope with the ever-increasing demands of procurement. This would inevitably lead to a delay to treatment or an inability to provide fertility preservation.

There are numerous obvious advantages to children being treated locally. In the initial phase, patients recently diagnosed with a potentially life-threatening illness, had to travel sometimes very long distances for fertility treatment away from their primary care team and family support. In contrast locally, the peri-operative recovery period is optimised in familiar surroundings when questions or clinical queries can be answered by the local team. Surgery may also require pre-operative investigations which can be performed locally and the results readily available. This also avoids lengthy handovers for complex patients with the potential introduction of errors. Different trusts also use specific brands of equipment and therefore staying local ensures that the potential for clinical incidents such as incorrect line insertion are minimised.

Given the increasing demand for this service, this model utilises resources efficiently and prevents procurement congestion at the Hub. It must be noted however that the Hub site tissue bank remains busy as they are still required to process all specimens received; regardless of procurement site. Integrating multiple centres within the system encourages co-operative working putting the patient at the centre of care.

However, there are also several key challenges faced throughout the expansion. First and foremost, there are a number of differences in systems and processes across the centres which lead to variability in terms of coordination and booking of lists. Our Hub has dedicated cryopreservation lists, which are elective theatre lists ringfenced for procurement. Our wider multidisciplinary team includes interventional radiology, paediatric haematology and oncology, and paediatric general surgery to ensure children booked for fertility preservation surgery have all procedures carried out under a single general anaesthetic. All staff members are consistent and procurement is performed by a select group of surgeons using standardised equipment and operative technique. Due to the frequency of these lists and their coordination, patients can be accommodated at late notice.

Within spoke centres, however, the processes are more varied with some centres having a nominated surgeon and dedicated operating time and others having to rely on ad hoc arrangements. When surgeons have to rely upon using emergency lists there are issues with cancellation of cases due to overriding emergencies and delays in the patient’s definitive treatment. Furthermore, vital resources such as the transport of specialist tissue storage equipment by courier then also becomes futile and must be rearranged.

A recent publication has demonstrated that the use of the SILS technique in comparison to standard multiport laparoscopic surgery has resulted in the harvest of an increased number of ampules of ovarian tissue for cryopreservation [7]. The Hub is the only centre that uses this technique, A recent survey of all 18 centres in England and Wales that provide ovarian procurement stated that 82% still use conventional 3-port laparoscopy [13]. We also use Ligasure in order to minimise thermal injury. However, there is variability in surgical technique across all Spoke sites. Electro-cauterisation techniques in procurement are vital to ensuring good quality specimens and the OCTB is now prospectively collecting data on thermal damage and the surgical technique used. With respect to testicular cryopreservation, surgical techniques have led to a discrepancy in the size of specimens.

We have recognised the need to develop a central administrative structure and better liaison and communication with our allied centres. Aims of our future project will include attempting to identify an optimal technique and unify this across the UK to optimise tissue quality. To add to this, standardisation across all centres would lead to the hope of providing a similar patient experience for every child who undergoes fertility preservation surgery. This will enhance the ability of the model to adapt as changes occurs. This idea will provide a framework for further expansion, as the demand increases further. The uniqueness of our article is that this is the first description of a paediatric and adolescent network within the United Kingdom.

## Conclusions

Through using a Hub and Spoke model, we have provided fertility preservation to an ever-growing population of children and young adults at high risk of infertility. Further work is required to decrease variability within our system. We aim to begin this process by prospectively surveying the centres to benchmark current practice. We will use this data to attempt to standardise equipment and unify surgical technique so that specimens are of high quality.
